# Inflammatory Myeloradiculitis Secondary to Pembrolizumab: A Case Report and Literature Review

**DOI:** 10.1155/2020/8819296

**Published:** 2020-08-18

**Authors:** M. L. Vickers, B. Seidl, K. Bigby, B. Chern, L. Eriksson, G. Hartnett, C. Gericke, R. Chew

**Affiliations:** ^1^Faculty of Medicine, University of Queensland, Brisbane, Queensland, Australia; ^2^Biomedical Engineering and Clinical Sciences, Queensland University of Technology, Brisbane, Australia; ^3^Department of Medical Oncology, Redcliffe Hospital, Redcliffe, Queensland, Australia; ^4^Herston Health Sciences Library, University of Queensland, Brisbane, Queensland, Australia; ^5^Department of Neurology, The Prince Charles Hospital, Brisbane, Queensland, Australia; ^6^Infectious Disease, Redcliffe Hospital, Redcliffe, Queensland, Australia

## Abstract

Immune checkpoint inhibitors are the most important new medications in oncology and include inhibitors of programmed cell death protein-1 (PD-1) such as Pembrolizumab, Nivolumab, and Cemiplimab. These anticancer agents prevent tumour immune evasion and have been associated with a range of immune-related adverse events (irAEs) including those involving the nervous system. In this case report and literature review, we present the first case of inflammatory myeloradiculitis secondary to Pembrolizumab. We also summarise the characteristics, treatment, and outcomes of other cases reported in the literature which include a component of myelitis. Finally, we make general recommendations on management.

## 1. Introduction

We report the first case of inflammatory myeloradiculitis following administration with the immune checkpoint inhibitor (ICI) Pembrolizumab. The development of ICIs has been the most notable advancement in cancer care in recent years. These medications act on immune inhibitory receptors including the cytotoxic T lymphocyte-associated antigen 4 (CTLA-4) and the programmed cell death protein-1 (PD-1) and its ligand (PD-L1) [1, 2]. The PD-1 receptor is an important pharmacological target because it inhibits T-lymphocyte proliferation and survival, induces apoptosis of tumour cells, and promotes differentiation of CD4+ T cells [3]. Inhibition of PD-1 limits tumour immune evasion by effectively taking the brakes off the immune system. In Australia, PD-1 inhibitors in common clinical use include Pembrolizumab and Nivolumab, which are approved for the treatment of multiple cancers including bladder and urothelial cancers, non-small-cell lung cancer, melanoma, lymphoma, and head and neck cancers [4]. The newer agent, Cemiplimab, is currently approved in the United States for use in squamous cell carcinoma and is undergoing clinical trial for further indications.

With the rapidly expanding use of ICIs such as Pembrolizumab, we have also seen growing recognition of the potential for immune-related adverse events (irAEs) [5]. These can impact any organ or body system and the more commonly encountered irAEs including hepatitis, colitis, pneumonitis, endocrine dysfunction, and dermatological disorders. In addition, neurological complications have been reported with a frequency of up to 4% and include encephalopathies, polyradiculopathies, Guillain-Barré-like syndromes, and myasthenic syndromes [6–15]. In order to summarise the relevant features, treatment, and outcomes of other myelitis cases with or without associated syndromes, we carried out a thorough systematic review of the literature and report the findings.

## 2. Case Presentation

A 73-year-old man presented to the emergency department with right leg swelling and an acute kidney injury. CT scans revealed a 6.2 mm unresectable right pelvic mass compressing the external iliac vein and ureters, a right acetabular metastasis, and external iliac vein thrombosis. Subsequent bladder biopsy led to the diagnosis of locally advanced transitional cell carcinoma. Malignant cells were positive for CK7, GATA3, and p40 with patchy positive staining for CK20. The patient had a background of benign prostatic hypertrophy, stage three chronic kidney disease, and melanoma of the right shin which had been excised several months prior. During a three-month period, he was treated with three cycles of gemcitabine on days one and eight in a 21-day cycle. Due to the lack of tumour response and multiple side effects, this was ceased, and one month later, he was commenced on Pembrolizumab. He received three 200 mg doses over a nine-week period. Three days prior to the final dose, he developed acute on chronic lumbar back pain with no inciting trauma. Over the following week, he developed an asymmetrical grade 3 paraesthesia of the upper and lower limbs, though retained brisk reflexes [16]. He also developed hypoesthesia and hypoalgesia in a banded distribution extending from the T10 to L1 dermatomes. Pembrolizumab was discontinued, and neurooncological investigations commenced. A Naranjo score of 7 was calculated, indicating a probable adverse drug reaction.

Brain and spine magnetic resonance imaging (MRI) demonstrated no evidence of haemorrhagic, ischaemic, or metastatic events and no cord compression. An inversion sequence could not be administered due to significant renal impairment. Fat saturation sequencing demonstrated no evidence of radiculopathy. Nerve conduction studies demonstrated bilaterally reduced lower limb motor units with retained sensory responses and reduced F-waves in the upper limbs. F-waves compare the conduction in the proximal half of the nerve pathway to the distal and can be used to distinguish a root lesion from a distal neuropathy [17]. Nerve conduction findings assisted in the exclusion of axonal loss and demyelinating syndromes such as acute inflammatory demyelinating polyneuropathy. Cerebrospinal fluid (CSF) was clear with an elevated protein level (2100 mg/L, normal 150-500 mg/L) and raised white blood cell count (35 × 10 [6]/L). There were no malignant cells or organisms, and the glucose level was normal (3.2 mmol/L). Multiple kappa and lambda IgG bands were present in trace amounts. Additional laboratory tests for anti-acetylcholine receptor antibodies, anti-ganglioside antibodies, and an infectious screen were negative. Respiratory function tests were also performed and normal.

Treatment included a course of intravenous immunoglobulin (IVIG) commenced at 30 g then 25 g daily for five days. The patient had been receiving 2 mg daily of dexamethasone for treatment of cancer symptoms, and this was left unchanged. Mild improvement in symptoms occurred one week following IVIG, and the patient was then transferred to a Rehabilitation Unit. After five weeks of multidisciplinary team input, he was discharged able to walk with a single point stick. A restaging CT scan was performed which demonstrated progression of acetabular cortical bone destruction adjacent to the malignancy compared to four months earlier, but no new metastatic disease or lymphadenopathy. No further immune treatment was offered for the primary malignancy.

## 3. Discussion

**Figure 1:**
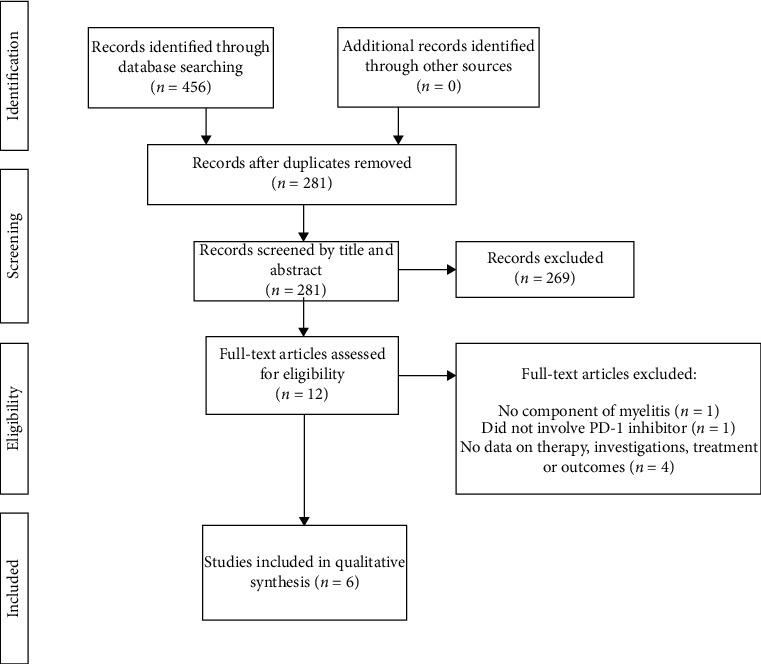


We aimed to describe a rare case of inflammatory myeloradiculitis following Pembrolizumab therapy and to review the literature for similar cases of myelitis with or without associated features. Using predefined terms, a systematic review of the literature was performed with PubMed, Embase, Web of Science, Scopus, Cochrane Central, and CINAHL from inception to 24^th^ March 2020. Results were screened and included if they reported myelitis following treatment with any PD-1 inhibitor. A total of ten cases were identified [18–27]. Four cases were not included because the authors provided no data on the patient, investigations, treatment, or outcomes [18, 19, 22, 26]. Two cases reported myelitis in the context of associated radiotherapy [20, 21]. All cases received steroid therapy [20, 21, 23–25, 27]. Four cases received plasmapheresis [21, 23, 24, 27], and two received intravenous immunoglobulin [21, 27]. Four of the six included cases demonstrated significant improvement in their symptoms [20, 21, 23, 27]. A summary of reported myelitis cases secondary to PD-1 inhibitor therapy is provided in Table 1. A summary of treatment and outcomes is provided in Table 2. A list of search terms are provided in Appendix A. A PRISMA flow chart of the case inclusion process is shown in Figure 1, and search results by database are provided in Table 3.

There are not enough cases in the literature to form specific management recommendations in the treatment of myelitis with or without associated syndromes in the context of PD-1 inhibitor therapy. Based on our experience, however, we suggest early interruption of the offending agent, neurological consult, and baseline investigations including inflammatory markers, autoimmune screen, nerve conduction studies, CSF analysis, and spinal MRI. We note that while all included cases used steroid therapy in their management, there was no clear consensus on the dose or regimen of therapy. Similarly, there were varied approaches to treatment with IVIG and plasmapheresis. We agree with Möhn et al. who suggests that clinicians should have a low threshold for early initiation of these modalities when neurological irAEs occur in the context of ICI therapy [28].

Predicting the response to PD-1 inhibitor therapy is problematic. This is due to the lack of a single reliable biomarker, varied success based on cancer subtype and the complexity of factoring in patient tumour burden, lymphocyte infiltration, comorbidities, and baseline performance status [29, 30]. Despite this, in some patients, PD-1 inhibitors have been shown to dramatically improve outcomes and are also generally better tolerated than chemotherapy with multiple studies reporting lower rates of neutropenia, thrombocytopenia, anaemia, fatigue, nausea, gastrointestinal toxicity, and neuropathy [31, 32]. While clinical trials largely exclude participants at high risk of adverse events, the growing role of these agents in daily clinical practice has broadened their application and uncovered a number of potentially serious neurological irAEs. This case report of myeloradiculitis secondary to Pembrolizumab highlights one such example and the importance of early recognition and treatment.

## Figures and Tables

**Table 1:** Reported cases and findings of myelitis-related adverse events secondary to PD-1 inhibitor therapy.

Author	Diagnosis	Case	Immune checkpoint inhibitor	Investigations and clinical findings
Carausu et al. [20]	Radiation myelitis	68 male, metastatic non-small-cell lung cancer	Pembrolizumab 8 cycles over 24 weeks	Spine MRI T1 hypointense signal and enhancement at site of prior radiation (30 Gray in 10 fractions), CSF protein 0.84 g/L
				Left lower limb muscle weakness, paraesthesia, difficulty urinating, rapid bowel movements
Chang et al. [21]	Transverse myelitis	68 male, metastatic melanoma	Combination ipilimumab and Nivolumab 3 cycles, then 1 dose Pembrolizumab	T5 to T10 enhancement at site of prior radiation (30 Gray in 10 fractions to T7-T10), CSF protein 99 mg/dL
				Intermittent tingling and numbness at soles of feet with ascension to knees during 2 months of Nivolumab, rapid progression after Pembrolizumab to gait instability, ataxia, ascension of sensory loss to hips, urinary retention, and faecal incontinence
Durães et al. [23]	Encephalomyelitis	58 female, metastatic melanoma	Pembrolizumab 2 cycles	MRI T2 hyperintense lesions with gadolinium enhancement, CSF protein 292 mg/dL, oligoclonal IgG bands
				Left hemiparesis, global hyporeflexia, distal hypoesthesia, and gate impairment without ataxia
Narumi et al. [24]	Neuromyelitis optica spectrum disorder	75 male, squamous cell carcinoma of lung	Nivolumab 1 dose	T2 hyperintense lesions C5-C6 and Th12-L1, CSF white cell count 1195 microL, CSF protein 381 mg/dL, and serum anti-aquaporin-4 antibody positive
				Acute paralysis lower limbs, sensory loss Th10 down, and urinary retention
Poretto et al. [25]	Myelitis	73 male, clear cell renal carcinoma	Nivolumab	Spine MRI demonstrated intensive contrast-enhancing intramedullary lesion with surrounding T2 hyperintensity, CSF protein 80 mg/dL
				Left lower limb hypoesthesia and urinary incontinence
Wilson et al. [27]	Longitudinally extensive transverse myelitis	35 male, classical Hodgkin lymphoma	Pembrolizumab 2 cycles 3 weeks apart	MRI myelitis from pons to lower thoracic spine, CSF demonstrated 24 mononuclear cells/m^3^, antihuman-IgG antibody bound to Tregs with high CD62L and CD25
				Acute urinary retention, constipation, spastic tetra paresis, and profound sensory level loss

**Table 2:** Treatment and outcomes of reported cases of myelitis-related adverse events secondary to PD-L1 inhibitor therapy.

Author	Treatment	Outcome
Carausu et al. [20]	Oral steroid 60 mg/day tapered over 2 months	Complete resolution, Pembrolizumab reinitiated without recurrence of myelitis
Chang et al. [21]	Dexamethasone 8 mg BD, bevacizumab 2 doses, 1000 mg daily methylprednisolone for 5 days, plasmapheresis, cyclophosphamide 100 mg/m3, and infliximab	Dramatic improvement post infliximab, however succumbed to malignancy soon after
Durães et al. [23]	Corticosteroid therapy for 5 days and plasma exchange for 7 sessions	Almost complete symptomatic recovery with residual mild sensory complaints
Narumi et al. [24]	Pulse steroid therapy, plasmapheresis	Minimal improvement
Poretto et al. [25]	High-dose steroid	Mild clinical and radiological improvement
Wilson et al. [27]	Intravenous methylprednisolone followed by oral prednisolone taper, intravenous immunoglobulins, and plasma exchange	Near complete remission, mild residual hypertonia

**Table 3:** 

Database	Results
PubMed	65
Embase	234
Web of Science	36
Scopus	107
Cochrane Central	4
CINAHL	10
Total	456
After duplicates removed	281

## Data Availability

This is a single patient case report with data accessed through the electronic medical record system. Information on other cases discussed is freely accessible in the public domain.

## Appendix

### A. List of Search Terms

Pembrolizumab, nivolumab, cemiplimab, lambrolizumab, keytruda, MK-3475, SCH 900475, PD-1 inhibitor, opdivo, ONO-4538, BMS-936558, MDX-1106, Nivo, Libtayo, REGN2810, SAR439684 and myelopathy, myelopathies, myelitis, transverse myelitis, polyradiculoneuropathy, polyradiculoneuropathies, polyneuropathy, radiculopathy, polyneuropathies, radiculopathies, myeloradiculitis, polyradiculitis, polyneuropathies, and spinal cord diseases.
